# LncRNA NORAD Enhances Inflammatory Injury in Sepsis‐Associated Acute Lung Damage Through miR‐150‐5p/STAT1‐Dependent NF‐κB Activation

**DOI:** 10.1002/kjm2.70191

**Published:** 2026-03-03

**Authors:** Han Liu, Xi‐Xi Chen, Gui‐Hua Wei, Feng Li, Hai‐Yan Zong

**Affiliations:** ^1^ Intensive Care Unit, Jiujiang City Key Laboratory of Cell Therapy Jiujiang No. 1 People's Hospital Jiujiang Jiangxi China; ^2^ Department of Emergency, Jiujiang City Key Laboratory of Cell Therapy Jiujiang No. 1 People's Hospital Jiujiang Jiangxi China

**Keywords:** bronchial epithelial cells, miR‐150‐5p, NORAD, sepsis‐induced acute lung injury, STAT1

## Abstract

Acute lung injury (ALI) is a severe complication of sepsis, yet the role of lncRNA NORAD in its pathogenesis remains unclear. Using LPS‐stimulated BEAS‐2B and HBEC3‐KT cells as well as cecal ligation and puncture (CLP)‐induced ALI models in C57BL/6 mice, we found that NORAD expression was markedly upregulated and promoted cell injury characterized by reduced viability, enhanced apoptosis, increased cytokine secretion, and aggravated lung damage. Mechanistic studies combining dual‐luciferase and RIP assays revealed that NORAD directly bound miR‐150‐5p to derepress STAT1, leading to elevated STAT1, p‐STAT1, and p‐p65 levels and activation of JAK/STAT and NF‐κB signaling. Silencing NORAD alleviated lung injury in vitro and in vivo, as evidenced by improved cell survival, decreased concentration of total protein in bronchoalveolar lavage fluid (BALF), reduced inflammatory response, lower wet/dry (W/D) ratio, diminished caspase‐3 levels, and decreased histological injury scores in CLP mice. Furthermore, knockdown of miR‐150‐5p or overexpression of STAT1 abolished the protective effects of NORAD inhibition, whereas overexpression of miR‐150‐5p or knockdown of STAT1 mitigated the injury‐promoting effects of NORAD overexpression. These findings demonstrate that NORAD exacerbates sepsis‐induced ALI via the miR‐150‐5p/STAT1 axis, providing new insights into potential therapeutic targets for sepsis‐related lung injury.

## 1 Introduction

Sepsis arises from a dysregulated host response to microbial infection and represents a critical cause of morbidity and mortality worldwide [1]. Among its many complications, acute lung injury (ALI) is one of the most devastating outcomes, manifesting as acute respiratory failure with profound disturbances in pulmonary structure and function [2]. Sepsis‐induced ALI involves excessive activation of inflammatory cascades and cell death programs, leading to compromised alveolar epithelial barrier integrity, increased vascular and epithelial permeability, and accumulation of protein‐rich edema in the alveolar space [3, 4, 5]. Although canonical inflammatory signaling pathways, including NF‐κB and JAK/STAT, have been shown to amplify these pathological processes [6, 7, 8], the upstream regulatory mechanisms that control these pathways and their contribution to epithelial injury and cell fate decisions remain largely undefined.

Long noncoding RNAs (lncRNAs) are endogenous transcripts > 200 nucleotides that lack protein‐coding capacity but exert critical regulatory functions in disease‐related gene expression [9]. Emerging evidence suggests that lncRNAs modulate inflammatory responses by regulating cytokine expression in sepsis‐associated lung injury. For instance, Qiu et al. reported that lncRNA TUG1 alleviates sepsis‐induced pulmonary injury, apoptosis, and inflammation through the miR‐34b‐5p/GAB1 pathway [10], while Zhu et al. showed that lncRNA CASC2 protects alveolar epithelial cells from septic injury via the miR‐152‐3p/PDK4 axis [11]. In contrast, Liu et al. demonstrated that lncRNA NEAT1 exacerbates lung injury by suppressing angiotensin‐converting enzyme 2 (ACE2) in models of sepsis‐induced acute respiratory distress syndrome (ARDS) [12]. Among these lncRNAs, noncoding RNA activated by DNA damage (NORAD) has attracted particular interest due to its regulatory roles in lipid metabolism, fibrosis, and inflammation [13, 14]. Elevated NORAD expression has been observed in neonatal sepsis patients, and its suppression reduced LPS‐driven inflammatory responses [15]. Consistently, Xie et al. found that NORAD knockdown attenuated kidney injury and limited inflammation and apoptosis in LPS‐stimulated HK‐2 cells via the miR‐577/GOLPH3 axis [16]. Despite these insights, the role and molecular mechanisms of NORAD in sepsis‐induced ALI remain largely unexplored.

MicroRNAs (miRNAs) are small, highly conserved noncoding RNAs of approximately 18–24 nucleotides that regulate gene expression by binding to complementary sequences in target mRNAs, thereby influencing diverse cellular processes [17]. Increasing evidence indicates that miRNAs modulate apoptosis and inflammatory cytokine release during sepsis progression [18]. Among them, miR‐150‐5p has been shown to be markedly downregulated in patients with chronic obstructive pulmonary disease (COPD), where it also serves as a potential diagnostic and prognostic biomarker [19]. In patients with ARDS, miR‐150‐5p downregulation was associated with increased apoptosis of human pulmonary microvascular endothelial cells (HPMECs), elevated proinflammatory cytokine expression, and aggravated lung injury in animal models, whereas its restoration suppressed these pathological changes [20]. The Janus kinase/signal transducer and activator of transcription (JAK/STAT) pathway is a key mediator of cytokine signaling and immune regulation in sepsis [21]. STAT1, a central transcription factor in this pathway, is required for LPS‐induced gene expression and has recently been reported to promote sepsis‐associated inflammatory lung injury in ARDS [22]. Furthermore, inhibition of STAT1/3, along with NF‐κB and MAPK, contributes to the protective effects of Harmine in sepsis‐induced cardiomyopathy [23]. Interestingly, NORAD has also been shown to modulate the STAT3/STAT1 balance and regulate innate immune responses [24]. Bioinformatic predictions suggest that NORAD may interact with miR‐150‐5p, with STAT1 identified as a downstream target of miR‐150‐5p. Based on these findings, we hypothesize that NORAD regulates inflammatory responses in sepsis‐induced ALI through the miR‐150‐5p/STAT1 axis.

The present study aimed to elucidate the expression pattern and regulatory role of the NORAD/miR‐150‐5p/STAT1 axis in LPS‐stimulated cell cultures and a murine cecal ligation and puncture (CLP) model of sepsis‐induced ALI, thereby providing potential therapeutic targets and a theoretical basis for future treatment strategies.

## 2 Materials and Methods

### 2.1 Cell Culture and Treatment

Two human bronchial epithelial cell lines, BEAS‐2B (CRL‐3588) and HBEC3‐KT (CRL‐4051), were obtained from the American Type Culture Collection (ATCC, Manassas, VA, USA). BEAS‐2B cells were maintained in Dulbecco's Modified Eagle's Medium (DMEM; Sigma‐Aldrich), while HBEC3‐KT cells were cultured in Ham's F‐12 K medium. Both media were supplemented with 10% fetal bovine serum (FBS), 100 U/mL penicillin, and 100 μg/mL streptomycin (Gibco). Cells were incubated at 37°C in a humidified atmosphere containing 5% CO_2_. To establish an in vitro model of sepsis‐induced ALI, cells were treated with 100 ng/mL lipopolysaccharide (LPS; cat. no. L2630) for 24 h.

### 2.2 Cell Transfection

Lentiviral vectors carrying small hairpin RNAs (shRNAs) targeting NORAD (sh‐NORAD), STAT1 (sh‐STAT1), miR‐150‐5p mimics, inhibitor, and their corresponding negative controls (NCs) were obtained from GenePharma (Shanghai, China). For NORAD and STAT1 overexpression, full‐length sequences were cloned into the pcDNA3.1 vector by Obio Company (Shanghai, China) to generate OE‐NORAD and OE‐STAT1 plasmids, with the empty pcDNA3.1 vector (OE‐NC) serving as the control. Transfection was performed using Lipofectamine 3000 (Invitrogen, Carlsbad, CA, USA) when BEAS‐2B and HBEC3‐KT cells reached approximately 80% confluence. Post‐transfection (48 h), cells were challenged with 100 ng/mL LPS for 24 h prior to further experimentation.

### 2.3 Dual‐Luciferase Reporter Assay

The potential binding sites between NORAD and miR‐150‐5p, as well as between miR‐150‐5p and STAT1, were predicted using the StarBase (http://starbase.sysu.edu.cn/) and TargetScan (https://www.targetscan.org/vert_71/) databases, respectively. Wild‐type (NORAD‐WT and STAT1‐WT) and mutant (NORAD‐MUT and STAT1‐MUT) reporter plasmids were generated by amplifying and cloning the corresponding wild‐type or mutated binding sequences into the pmirGLO luciferase vector (Promega, Madison, WI, USA). These constructs were then co‐transfected into BEAS‐2B cells along with miR‐150‐5p inhibitor or NC inhibitor, and into HBEC3‐KT cells with miR‐150‐5p mimics or NC mimics, using Lipofectamine 3000 (Invitrogen). After 48 h, cell supernatants were collected, and luciferase activity was measured using the Dual‐Luciferase Reporter Assay System (Promega). Relative luciferase activity was expressed as the ratio of *firefly* to *Renilla* luciferase activity.

### 2.4 RNA Immunoprecipitation (RIP) Assay

RIP assays were conducted in BEAS‐2B and HBEC3‐KT cells using the Magna RIP RNA‐Binding Protein Immunoprecipitation Kit (Millipore, No. 03‐110; Bedford, MA, USA). Briefly, cells were harvested, centrifuged, and lysed in RIP lysis buffer. The resulting lysates were incubated overnight at 4°C with magnetic beads conjugated to either anti‐Ago2 or control IgG antibodies. Immunoprecipitated RNAs were then purified, and the expression levels of NORAD and miR‐150‐5p were quantified by real‐time PCR in triplicate.

### 2.5 Cell Viability Assay

Cell viability of BEAS‐2B and HBEC3‐KT cells was assessed at 24, 48, and 72 h using the Cell Counting Kit‐8 (CCK‐8; Dojindo, Japan). Cells (5 × 10^3^/well) were seeded into 96‐well plates and cultured at 37°C with 5% CO_2_. At each time point, 10 μL of CCK‐8 solution was added to each well and incubated for 2 h. Absorbance at 450 nm was measured using a microplate reader (BioTek, USA).

### 2.6 Cell Apoptosis Assay

Apoptosis of BEAS‐2B and HBEC3‐KT cells was analyzed using the Annexin V‐Fluorescein Isothiocyanate (FITC)/Propidium Iodide (PI) Apoptosis Detection Kit (Cat# 556547, BD Biosciences, San Jose, CA, USA) following the manufacturer's protocol. Briefly, cells (3 × 10^5^ cells/well) were seeded into six‐well plates and incubated for 24 h. After harvesting by trypsinization and washing with cold PBS, cells were stained with Annexin V‐FITC and PI for 15 min at room temperature in the dark. Apoptotic cells were then analyzed by flow cytometry using a BD FACSCanto II cytometer (BD Biosciences, USA).

### 2.7 Establishment of Mouse Model and Experimental Groups

Adult male C57BL/6 mice (8–9 weeks old) were obtained from Beijing Vital River Laboratory Animal Technology Co. Ltd. (Beijing, China) and housed under specific pathogen‐free (SPF) conditions (22°C–24°C, 60% humidity, 12 h light/dark cycle) with free access to food and water. After 7 days of acclimatization, mice were randomly assigned to four groups (*n* = 6/group): sham, CLP, CLP + sh‐NC, and CLP + sh‐NORAD. For adeno‐associated virus (AAV) pretreatment, mice in the latter two groups received 20 μL of either AAV‐sh‐NC or AAV‐sh‐NORAD (1 × 10^7^ viral genomes [vg]/mL; Shandong WZ Biotechnology Co. Ltd.) via tail vein injection 7 days prior to CLP and were monitored for 48 h. CLP surgery was performed under anesthesia with 2% sodium pentobarbital (40 mg/kg ip). After shaving and disinfecting the abdomen, a midline incision was made to expose the cecum, which was ligated below the ileocecal valve and punctured once with a 22‐gauge needle. A small amount of feces was gently extruded before returning the cecum to the peritoneal cavity. The abdominal incision was then sutured, and sterile saline was administered subcutaneously every 6 h to maintain fluid balance. Sham‐operated mice underwent the same procedure without ligation or puncture. At 6 h post‐surgery, mice were euthanized using an overdose of 10% sodium pentobarbital. Bronchoalveolar lavage fluid (BALF) and lung tissues were collected, and pulmonary edema was assessed via the lung wet‐to‐dry weight ratio. All animal procedures were performed in accordance with national guidelines and approved by the Experimental Animal Ethics Committee of Jiujiang No. 1 People's Hospital (Jiangxi, China).

### 2.8 Hematoxylin–Eosin (H&E) Staining

Lung tissue morphology was assessed using standard H&E staining. Samples were fixed in 4% paraformaldehyde for 24 h, dehydrated, embedded in paraffin, and sectioned coronally at 5 μm thickness. Sections were stained following conventional H&E protocols, then dehydrated through graded alcohols, cleared with xylene, and mounted with neutral resin. Histological analysis was performed under an Olympus BX 53 microscope (Tokyo, Japan). Lung injury was scored based on hemorrhage, inflammation, and edema, using a semi‐quantitative scale: 0 (normal), 2 (mild), 4 (moderate), 6 (severe), and 8 (extremely severe).

### 2.9 Detection of Lung Wet‐to‐Dry (W/D) Ratio

The W/D ratio was determined to assess pulmonary edema severity. Immediately after euthanasia, the right upper lobe was excised, and surface exudates/blood were removed using filter paper. Wet weight (W) was recorded, followed by dehydration at 80°C for 48 h to obtain dry weight (D). The W/D ratio was then calculated.

### 2.10 Protein Concentration Quantification

BALF samples were centrifuged at 1500*g* for 10 min at 4°C to remove cellular debris. The protein concentration in the collected supernatant was then determined using a Bicinchoninic Acid (BCA) Protein Assay Kit (Pierce, Rockford, IL, USA), according to the manufacturer's instructions.

### 2.11 ELISA Assay

Concentrations of TNF‐α and IL‐1β in cell culture supernatants and mouse BALF were measured using species‐specific ELISA kits (TNF‐α: #ab181421 for human, #ab208348 for mouse; IL‐1β: #ab46052 for human, #ab197742 for mouse; Abcam, UK), following the manufacturer's instructions.

### 2.12 Quantitative Real‐Time PCR

Total RNA was extracted from cultured cells and tissue samples using TRIzol Reagent (Invitrogen, USA), following the manufacturer's protocol. For lncRNA analysis, complementary DNA (cDNA) was synthesized using the PrimeScript RT Reagent Kit (Cat# RR047A, Takara, Japan). For miRNA quantification, reverse transcription was performed using the miRNA First‐Strand cDNA Synthesis Kit (Cat# RR716, Takara, Japan). Quantitative real‐time PCR was conducted using either the TB Green Premix Ex Taq II (Tli RNaseH Plus; Cat# RR820A, Takara, Japan) for lncRNA detection or the miRcute miRNA qPCR Detection Kit SYBR Green (Cat# FP411, Tiangen Biotech, China) for miRNA analysis. GAPDH and U6 were used as endogenous normalization controls for mRNA and miRNA quantification, respectively. The thermal cycling conditions were as follows: initial denaturation at 95°C for 30 s, followed by 40 cycles of 95°C for 5 s and 60°C for 30 s. Relative gene expression was calculated using the 2^−ΔΔ*C*^_t_ method. All primers were purchased from OriGene (Rockville, MD, USA), and detailed sequences are provided in Table 1.

**TABLE 1:** Primers for quantitative real‐time PCR.

Gene	Forward (5′–3′)	Reverse (5′–3′)
Human NORAD	CCTAGGAGTGGTTGCATTTGG	TCTCCCTGCATACCTCCTCTG
Human miR‐150‐5p	TCTCCCAACCCTTGTAC	GAACATGTCTGCGTATCTC
Human GAPDH	GGAAGGTGAAGGTCGGAGTC	TCGCCCCACTTGATTTTGGA
Human U6	GGAAGGTGAAGGTCGGAGTC	TCGCCCCACTTGATTTTGGA
Mouse NORAD	TAACGGATTGGTAGGGTGGC	TGCACTTGTGTGGAGGCTAA
Mouse miR‐150‐5p	CTCCCAACCCTTGTACCA	GAACATGTCTGCGTATCTC
Mouse GAPDH	CTTCTCCTGCAGCCTCGT	ATGAAGGGGTCGTTGATGGC
Mouse U6	CTCGCTTCGGCAGCACA	AACGCTTCACGAATTTGCGT

### 2.13 Western Blot Analysis

Total protein was extracted from cultured cells and tissue samples using RIPA lysis buffer (Beyotime Biotechnology, China). Protein concentrations were measured using the Pierce BCA Protein Assay Kit (Thermo Scientific, USA). Equal amounts of protein (30 μg per sample) were resolved by 10% SDS‐PAGE and transferred onto polyvinylidene fluoride (PVDF) membranes (Millipore, USA). Membranes were blocked with 5% nonfat milk and incubated overnight at 4°C with the following primary antibodies: anti‐PCNA (1:1000, Cat# 13110, Cell Signaling Technology, USA), anti‐cleaved caspase‐3 (1:1000, Cat# 9661, CST), anti‐p‐JAK3 (1:1000, #AF8160, Affinity Biosciences), anti‐JAK3 (1:1000, A0748, ABclonal), anti‐p‐STAT1, anti‐STAT1 (1:10000, A19563, ABclonal), anti‐p‐p65 (1:10000, AP1294, ABclonal), anti‐p65 (1:5000, A19653, ABclonal), and anti‐GAPDH (1:5000, Cat# 10494‐1‐AP, Proteintech, USA). Following primary incubation, membranes were incubated with horseradish peroxidase (HRP)‐conjugated secondary antibodies (Santa Cruz Biotechnology, USA) for 2 h at room temperature. Protein bands were visualized using enhanced chemiluminescence (ECL) detection reagents (Cat# sc‐2048, Santa Cruz Biotechnology, USA) and quantified by densitometry using ImageJ software (NIH, USA).

### 2.14 Statistical Analysis

All statistical analyses were performed using GraphPad Prism 8.0. Data are presented as mean ± standard deviation (SD) from a minimum of three independent experiments. Comparisons between two groups were made using unpaired *t*‐tests, while one‐way ANOVA followed by Tukey's post hoc test was used for multiple group comparisons. A *p* < 0.05 was considered statistically significant.

## 3 Results

### 3.1 LncRNA NORAD Exacerbated LPS‐Induced Injury in Lung Epithelial Cells

**FIGURE 1:**
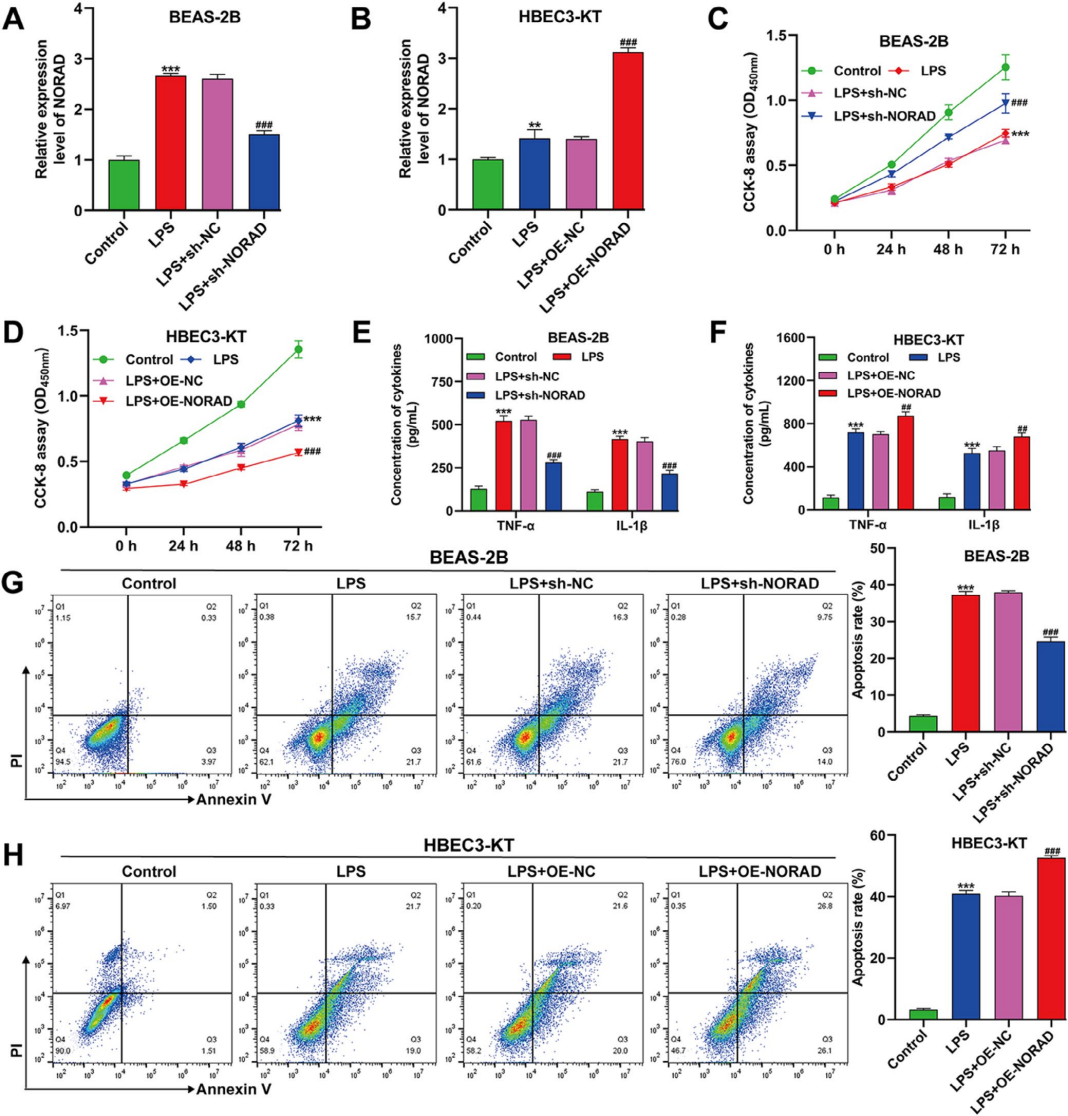
Effects of NORAD on LPS‐induced injury in lung epithelial cells. BEAS‐2B and HBEC3‐KT cells were transfected with sh‐NORAD or OE‐NORAD, respectively, followed by LPS exposure for 24 h. (A, B) NORAD expression was quantified by quantitative real‐time PCR to confirm transfection efficiency. (C, D) Cell viability was assessed using the CCK‐8 assay. (E, F) TNF‐α and IL‐1β levels in cell supernatants were measured by ELISA assay. (G, H) Cell apoptosis was evaluated via flow cytometry analysis. Data were presented as the mean ± SD. **p < 0.01, ***p < 0.001 versus control; ^##^ *p* < 0.01, ^###^ *p* < 0.001 versus LPS + sh‐NC or LPS + OE‐NC.

To investigate the role of lncRNA NORAD in sepsis‐induced ALI in vitro, BEAS‐2B cells (with relatively high endogenous NORAD expression) and HBEC3‐KT cells (with lower NORAD levels) were used to model LPS‐treated bronchial epithelial cells with NORAD knockdown or overexpression. Quantitative real‐time PCR confirmed successful transfection and modulation of NORAD expression (Figure 1A,B). The results of CCK‐8 assay demonstrated that NORAD knockdown significantly improved the viability of LPS‐treated BEAS‐2B cells (Figure 1C), whereas its overexpression further impaired viability in LPS‐treated HBEC3‐KT cells (Figure 1D). Consistently, silencing NORAD reduced the LPS‐induced secretion of proinflammatory cytokines TNF‐α and IL‐1β in BEAS‐2B cells (Figure 1E), while NORAD overexpression enhanced their levels in HBEC3‐KT cells (Figure 1F). Flow cytometry analysis revealed that NORAD knockdown significantly attenuated LPS‐induced apoptosis in BEAS‐2B cells (Figure 1G), whereas its overexpression exacerbated apoptosis in HBEC3‐KT cells (Figure 1H). Collectively, these results suggest that NORAD promotes LPS‐induced inflammatory injury and apoptosis in lung epithelial cells, thereby contributing to the pathogenesis of sepsis‐related ALI in vitro.

### 3.2 NORAD Directly Interacted With and Negatively Regulated miR‐150‐5p in Septic Lung Injury Models

**FIGURE 2:**
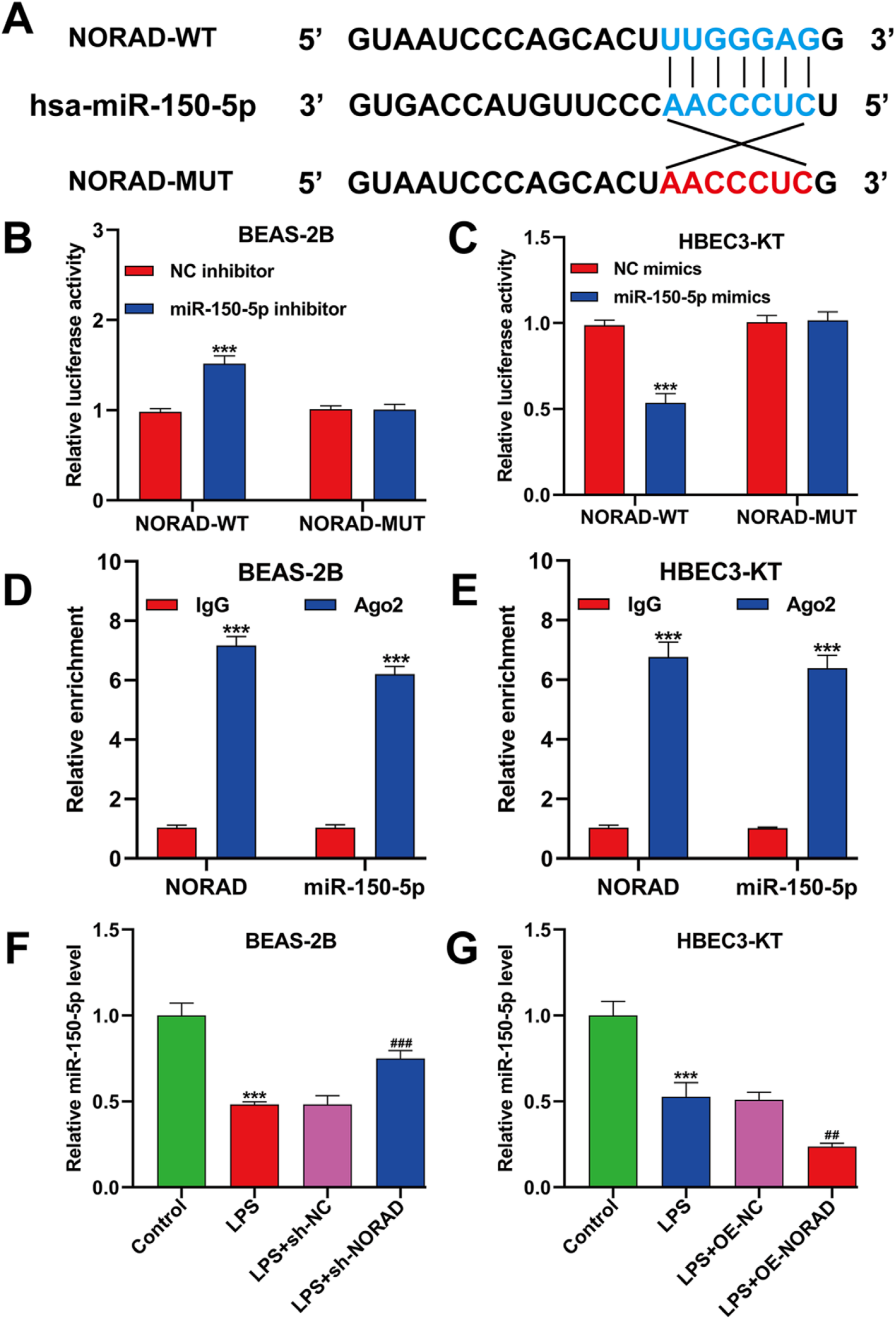
Experimental validation of the interaction between NORAD and miR‐150‐5p. (A) Bioinformatic prediction using the StarBase database showing a putative binding site of miR‐150‐5p within NORAD. (B) Dual‐luciferase reporter assay in BEAS‐2B cells showing higher luciferase activity in the NORAD‐WT group after co‐transfection with a miR‐150‐5p inhibitor, whereas no obvious change was observed in the NORAD‐MUT group. ***p < 0.001 versus NC inhibitor. (C) In HBEC3‐KT cells, miR‐150‐5p mimics decreased luciferase activity in the NORAD‐WT group but did not affect NORAD‐MUT. ***p < 0.001 versus NC mimics. (D, E) RIP analysis showing enrichment of NORAD and miR‐150‐5p in Ago2 immunoprecipitants compared with IgG controls in BEAS‐2B and HBEC3‐KT cells. ***p < 0.001 versus IgG. (F) Quantitative real‐time PCR analysis of miR‐150‐5p levels in BEAS‐2B cells after LPS stimulation with or without NORAD knockdown. (G) Quantitative real‐time PCR analysis of miR‐150‐5p levels in HBEC3‐KT cells after LPS stimulation with or without NORAD overexpression. ***p < 0.001 versus control; ^##^ *p* < 0.01, ^###^ *p* < 0.001 versus LPS + sh‐NC or LPS + OE‐NC. Data were presented as the mean ± SD.

Bioinformatic analysis using StarBase predicted miR‐150‐5p as a potential binding partner of NORAD (Figure 2A). This interaction was experimentally validated by dual‐luciferase reporter assays: in BEAS‐2B cells, co‐transfection with NORAD‐WT and a miR‐150‐5p inhibitor significantly increased luciferase activity compared with the NC, whereas the NORAD‐MUT construct abolished this effect (Figure 2B). Similarly, in HBEC3‐KT cells, miR‐150‐5p mimics markedly suppressed luciferase activity of NORAD‐WT but had no effect on NORAD‐MUT (Figure 2C). RIP assays further validated the association, showing enrichment of both NORAD and miR‐150‐5p in anti‐Ago2 complexes compared with IgG controls in BEAS‐2B (Figure 2D) and HBEC3‐KT cells (Figure 2E). In addition, LPS stimulation decreased miR‐150‐5p expression in lung epithelial cells. NORAD knockdown in BEAS‐2B cells restored miR‐150‐5p levels (Figure 2F), while NORAD overexpression in HBEC3‐KT cells further suppressed its expression (Figure 2G). Collectively, these findings demonstrate that NORAD directly binds to and negatively regulates miR‐150‐5p.

### 3.3 MiR‐150‐5p Attenuated NORAD‐Mediated Injury in LPS‐Induced Lung Epithelial Cells

**FIGURE 3:**
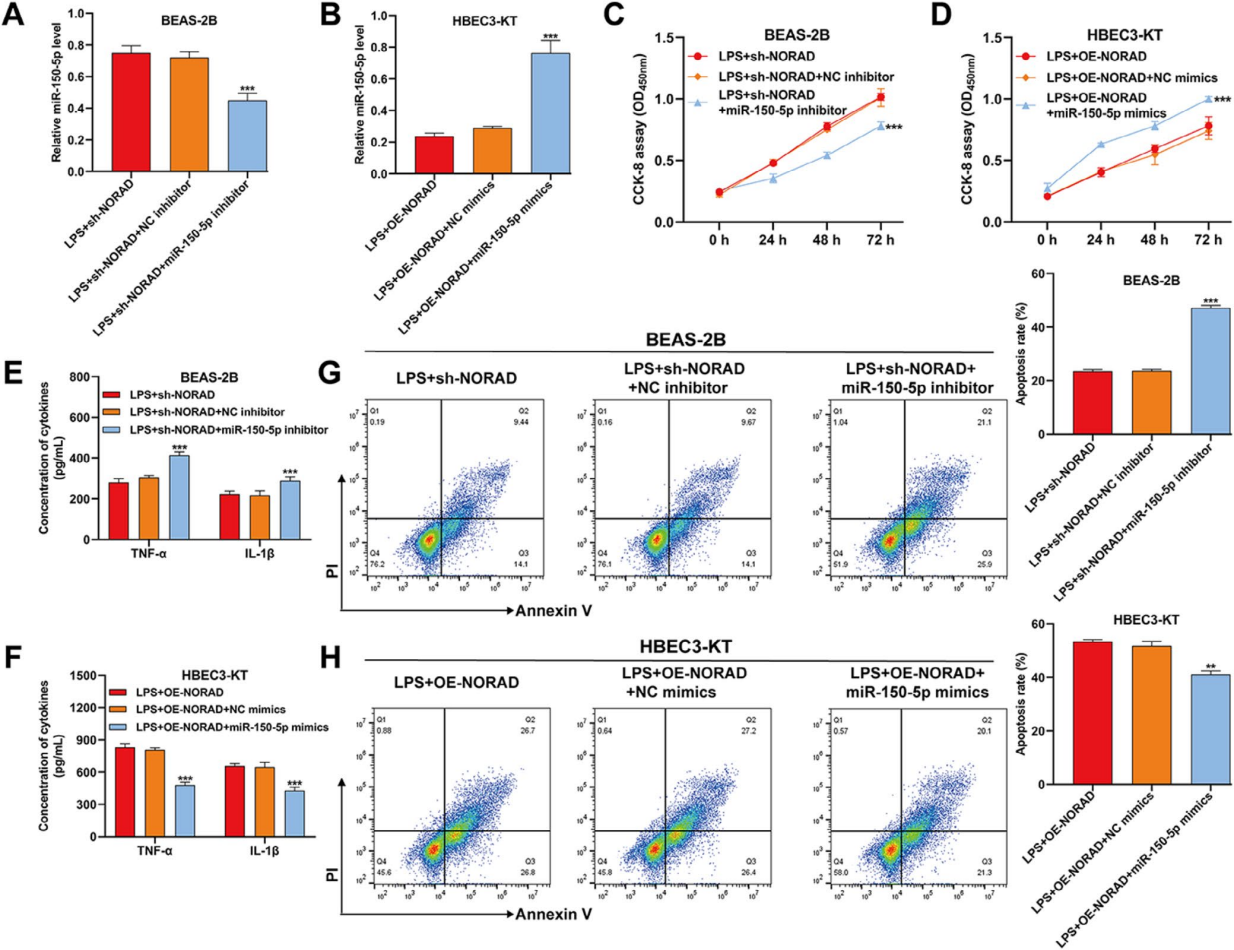
Influence of miR‐150‐5p on NORAD‐driven injury in LPS‐stimulated lung epithelial cells. BEAS‐2B cells were co‐transfected with sh‐NORAD and a miR‐150‐5p inhibitor, while HBEC3‐KT cells were co‐transfected with OE‐NORAD and miR‐150‐5p mimics, followed by LPS exposure for 24 h. (A, B) Quantitative real‐time PCR analysis of miR‐150‐5p expression after transfection. (C, D) Cell viability measured by CCK‐8 assay. (E, F) TNF‐α and IL‐1β levels in cell supernatants assessed by ELISA. (G, H) Cell apoptosis determined by flow cytometry. Data were presented as the mean ± SD. **p < 0.01, versus LPS + OE‐NORAD + NC mimics; ***p < 0.001 versus LPS + sh‐NORAD + NC inhibitor or LPS + OE‐NORAD + NC mimics.

To assess whether NORAD regulated LPS‐induced epithelial injury through miR‐150‐5p, rescue experiments were performed in BEAS‐2B cells co‐transfected with sh‐NORAD and a miR‐150‐5p inhibitor, and in HBEC3‐KT cells co‐transfected with OE‐NORAD and miR‐150‐5p mimics. Quantitative real‐time PCR confirmed that the inhibitor reduced miR‐150‐5p upregulation induced by NORAD knockdown in BEAS‐2B cells (Figure 3A), whereas the mimics restored miR‐150‐5p expression suppressed by NORAD overexpression in HBEC3‐KT cells (Figure 3B). CCK‐8 assays showed that the inhibitor decreased viability in sh‐NORAD‐treated BEAS‐2B cells, while the mimics enhanced viability in OE‐NORAD‐treated HBEC3‐KT cells (Figure 3C,D). ELISA results demonstrated that reduced TNF‐α and IL‐1β secretion caused by NORAD knockdown was reversed by miR‐150‐5p inhibition, whereas cytokine elevation induced by NORAD overexpression was alleviated by miR‐150‐5p overexpression (Figure 3E,F). Flow cytometry further revealed that miR‐150‐5p inhibition increased apoptosis in sh‐NORAD‐transfected BEAS‐2B cells, while miR‐150‐5p overexpression reduced apoptosis in OE‐NORAD‐transfected HBEC3‐KT cells (Figure 3G,H). Together, these findings indicate that NORAD aggravates LPS‐induced lung epithelial cell injury by downregulating miR‐150‐5p.

### 3.4 STAT1 Was Directly Regulated by miR‐150‐5p

**FIGURE 4:**
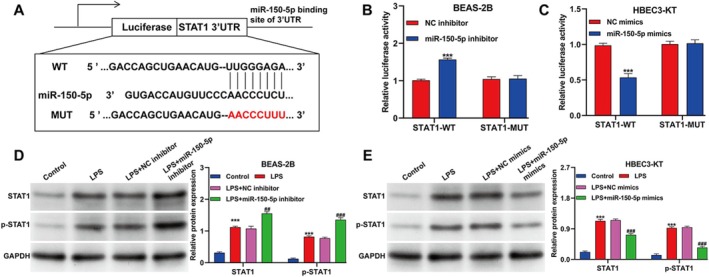
Identification of STAT1 as a target of miR‐150‐5p. (A) Bioinformatic prediction using TargetScan revealed that STAT1 3′‐UTR contains complementary binding sites for miR‐150‐5p. (B, C) Dual‐luciferase reporter assays demonstrated that co‐transfection of STAT1‐WT plasmid with miR‐150‐5p inhibitor significantly enhanced luciferase activity in BEAS‐2B cells, whereas co‐transfection with miR‐150‐5p mimics reduced luciferase activity in HBEC3‐KT cells. ***p < 0.001 versus NC inhibitor or NC mimics; (D) Western blot analysis showed that LPS stimulation increased STAT1 and p‐STAT1 expression in BEAS‐2B cells, which was further augmented by miR‐150‐5p inhibition. (E) Overexpression of miR‐150‐5p in HBEC3‐KT cells attenuated the LPS‐induced upregulation of STAT1 and p‐STAT1. Data were presented as the mean ± SD. ***p < 0.001 vs. LPS; ^##^ *p* < 0.01, ^###^ *p* < 0.001 versus LPS + NC mimics.

We next investigated the downstream regulatory mechanism of the NORAD/miR‐150‐5p axis in LPS‐induced lung epithelial cell injury. STAT1, a key component of the JAK/STAT signaling pathway involved in inflammatory regulation, was predicted by TargetScan to harbor the 3′‐UTR sequence complementary to miR‐150‐5p (Figure 4A). Dual‐luciferase reporter assays confirmed this interaction: luciferase activity was significantly enhanced in BEAS‐2B cells co‐transfected with STAT1‐WT and miR‐150‐5p inhibitor, whereas it was markedly suppressed in HBEC3‐KT cells co‐transfected with STAT1‐WT and miR‐150‐5p mimics (Figure 4B,C). Western blot analysis further demonstrated that LPS stimulation elevated both STAT1 and p‐STAT1 expression in BEAS‐2B cells, which was augmented by miR‐150‐5p inhibition (Figure 4D). Conversely, overexpression of miR‐150‐5p in HBEC3‐KT cells attenuated LPS‐induced upregulation of STAT1 and p‐STAT1 (Figure 4E). Collectively, these findings identify STAT1 as a direct downstream target of miR‐150‐5p.

### 3.5 STAT1 Was Involved in NORAD‐Mediated Modulation of LPS‐Induced Lung Epithelial Cell Injury

**FIGURE 5:**
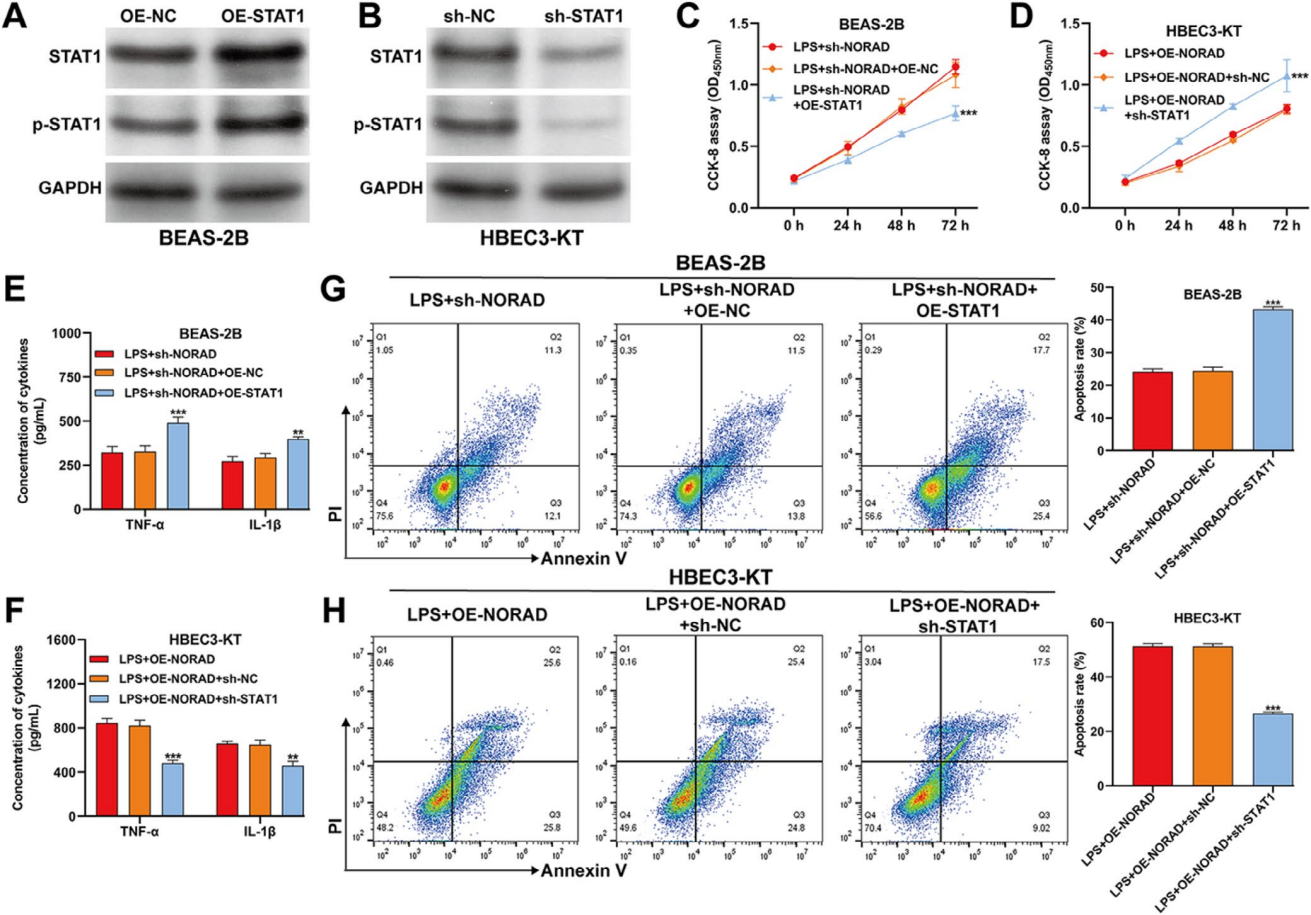
STAT1 mediates the regulatory effect of NORAD in LPS‐induced lung epithelial cell injury. (A) Western blot analysis showing STAT1 and p‐STAT1 overexpression efficiency in BEAS‐2B cells. (B) Western blot analysis confirming STAT1 and p‐STAT1 knockdown efficiency in HBEC3‐KT cells. (C, D) CCK‐8 assay showing the effects of STAT1 overexpression or knockdown on cell viability in response to NORAD modulation. (E, F) ELISA assays measuring TNF‐α and IL‐1β secretion under the indicated conditions. (G, H) Flow cytometry analysis of apoptosis rate in BEAS‐2B and HBEC3‐KT cells after NORAD and STAT1 manipulation. Data were presented as the mean ± SD. **p < 0.01, ***p < 0.001 versus LPS + sh‐NORAD + OE‐NC or LPS + OE‐NORAD + sh‐NC.

To investigate the involvement of STAT1 in the regulatory role of NORAD during LPS‐induced lung epithelial cell injury, we verified the successful overexpression of STAT1 and p‐STAT1 in BEAS‐2B cells (Figure 5A) and their efficient knockdown in HBEC3‐KT cells (Figure 5B). Functional assays demonstrated that STAT1 overexpression partially reversed the inhibitory effects of sh‐NORAD in BEAS‐2B cells, whereas STAT1 silencing mitigated the pro‐injury effects of NORAD overexpression in HBEC3‐KT cells. These changes were reflected in cell viability (Figure 5C,D), levels of proinflammatory cytokines TNF‐α and IL‐1β (Figure 5E,F), and apoptosis rate (Figure 5G,H), as assessed by CCK‐8, ELISA, and flow cytometry, respectively. Collectively, these findings indicate that NORAD aggravated LPS‐induced lung epithelial cell injury, at least in part, through STAT1 modulation.

### 3.6 NORAD Promoted LPS‐Driven STAT1 Phosphorylation and NF‐κB Signaling Through the miR‐150‐5p/STAT1 Axis

**FIGURE 6:**
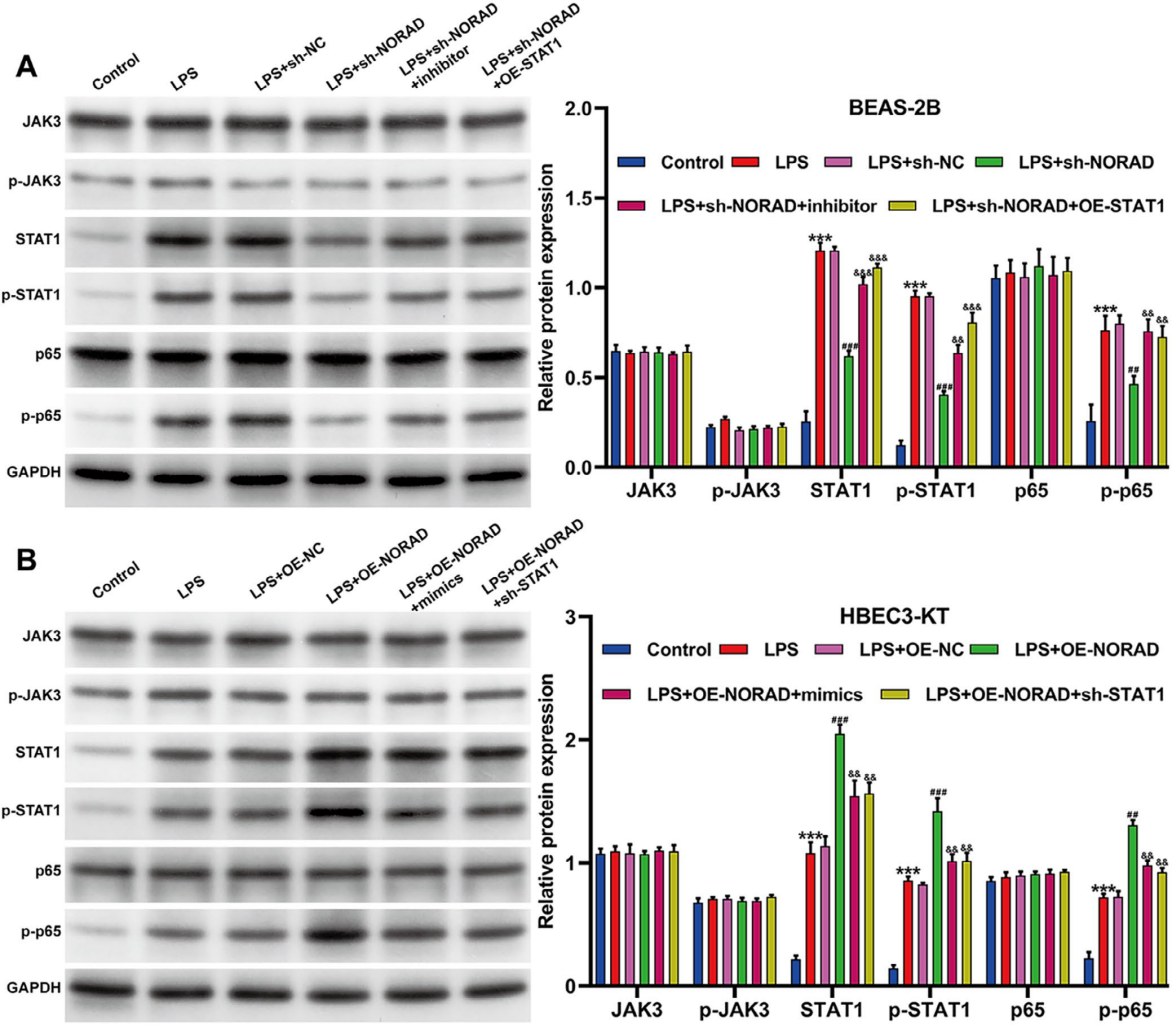
NORAD promoted LPS‐driven STAT1 phosphorylation and NF‐κB signaling through the miR‐150‐5p/STAT1 axis. (A) BEAS‐2B cells were treated with LPS and transfected with sh‐NORAD, sh‐NORAD + miR‐150‐5p inhibitor, or sh‐NORAD + STAT1 overexpression plasmid. Western blot analysis shows the protein levels of p‐JAK3, JAK3, p‐STAT1, STAT1, p‐p65, and p65 under different conditions. (B) HBEC3‐KT cells were treated with LPS and transfected with OE‐NORAD, OE‐NORAD + miR‐150‐5p mimics, or OE‐NORAD + sh‐STAT1. Western blot analysis shows the protein expression of p‐JAK3, JAK3, p‐STAT1, STAT1, p‐p65, and p65 in each group. Data were presented as the mean ± SD. ***p < 0.001 versus control; ^##^ *p* < 0.01, ^###^ *p* < 0.001 versus LPS + sh‐NC; ^&&^ *p* < 0.01, ^&&&^ *p* < 0.001 versus LPS + sh‐NORAD.

To clarify the role of NORAD in LPS‐driven STAT1 signaling and associated molecular responses, we conducted loss‐ and gain‐of‐function assays in BEAS‐2B and HBEC3‐KT cells. In BEAS‐2B cells (Figure 6A), LPS stimulation markedly increased the levels of p‐STAT1 and p‐p65, while JAK3 and p‐JAK3 levels remained largely unchanged. NORAD silencing (LPS + sh‐NORAD) significantly attenuated the LPS‐induced upregulation of p‐STAT1 and p‐p65, whereas co‐transfection with a miR‐150‐5p inhibitor or STAT1 overexpression plasmid (LPS + sh‐NORAD + inhibitor/OE‐STAT1) restored these changes, indicating that NORAD facilitates LPS‐induced STAT1 activation through the miR‐150‐5p/STAT1 axis. Similarly, in HBEC3‐KT cells (Figure 6B), NORAD overexpression (LPS + OE‐NORAD) further amplified LPS‐induced increases in p‐STAT1 and p‐p65 without affecting JAK3 or p‐JAK3, while co‐transfection with miR‐150‐5p mimics or STAT1 knockdown (LPS + OE‐NORAD + mimics/sh‐STAT1) blunted the response. Collectively, these findings demonstrate that NORAD promoted LPS‐induced STAT1‐mediated signaling by modulating the miR‐150‐5p/STAT1 axis, independent of JAK3 activation.

### 3.7 Knockdown of NORAD Ameliorated CLP‐Induced ALI In Vivo

**FIGURE 7:**
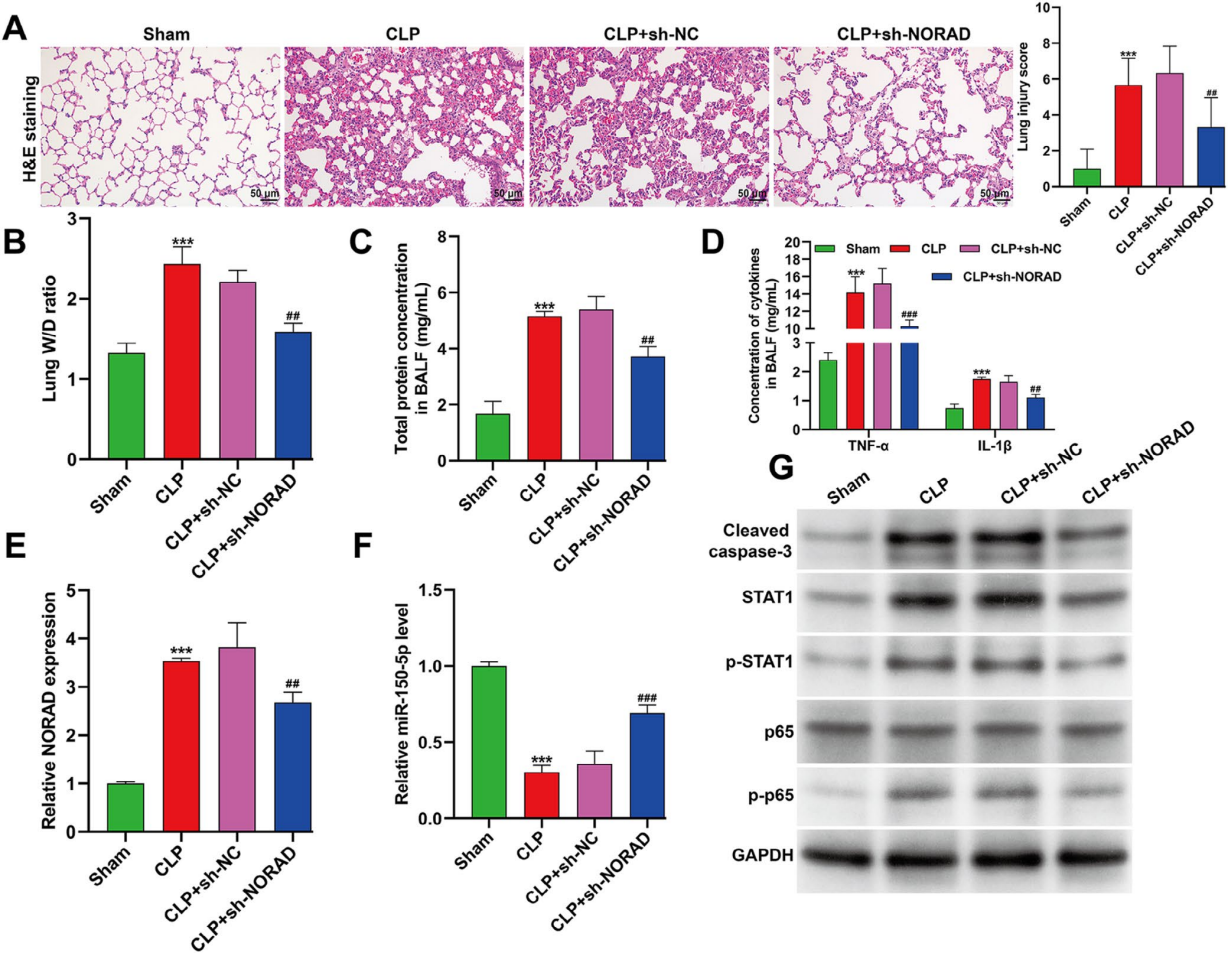
Knockdown of NORAD ameliorated CLP‐induced ALI in vivo. C57BL/6 mice were randomly assigned to three groups (*n* = 6 per group): Sham‐operated, CLP + AAV‐sh‐NC, and CLP + AAV‐sh‐NORAD. Mice in the latter two groups received tail vein injections of AAV‐sh‐NC or AAV‐sh‐NORAD prior to CLP to achieve in vivo NORAD knockdown. (A) Lung tissues were collected post‐euthanasia and subjected to H&E staining. Sham mice displayed normal alveolar architecture, whereas CLP mice exhibited alveolar collapse, congestion, interstitial and alveolar cell infiltration, thickened alveolar walls, and red blood cell accumulation. NORAD knockdown partially restored alveolar structure (Scale bar = 50 μm). Lung injury scores are shown. (B) Lung wet‐to‐dry (W/D) weight ratio was measured to assess pulmonary edema severity. (C) The total protein concentration was measured in BALF of mice. (D) TNF‐α and IL‐1β levels in bronchoalveolar lavage fluid (BALF) were assessed by ELISA assay. (E‐F) Quantitative real‐time PCR was performed to measure NORAD and miR‐150‐5p expression in lung tissues. (G) Western blotting evaluated cleaved caspase‐3, p‐STAT1/STAT1, and p‐p65/p65 levels. Data were presented as the mean ± SD. ***p < 0.001 versus sham; ^##^ *p* < 0.01, ^###^ *p* < 0.001 versus CLP + sh‐NC.

To investigate the role of NORAD in sepsis‐induced ALI, a CLP model was established in C57BL/6 mice. Mice were administered AAV‐sh‐NC or AAV‐sh‐NORAD via tail vein injection prior to CLP to achieve in vivo NORAD knockdown. Lung tissues were collected post‐euthanasia for H&E staining. Sham‐operated mice exhibited normal alveolar architecture, whereas CLP‐operated mice showed disrupted alveolar structure, congestion, collapse, interstitial and alveolar cell infiltration, thickened alveolar walls, and red blood cell accumulation, indicating severe lung injury. These pathological changes were attenuated in the CLP + sh‐NORAD group, with more intact alveolar structures and significantly lower lung injury scores compared with the CLP + sh‐NC group (Figure 7A). Consistent with these findings, the lung W/D weight ratio was markedly elevated in CLP mice relative to sham controls, while NORAD knockdown significantly reduced pulmonary edema (Figure 7B). In parallel, total protein concentrations in BALF were significantly increased in CLP‐treated mice, reflecting enhanced alveolar–capillary barrier permeability, and were significantly reduced following NORAD knockdown (Figure 7C). Furthermore, ELISA analysis demonstrated that NORAD knockdown significantly reduced the CLP‐induced elevations of proinflammatory cytokines TNF‐α and IL‐1β in BALF (Figure 7D). At the molecular level, quantitative real‐time PCR confirmed upregulation of NORAD in CLP lungs, which was effectively suppressed by sh‐NORAD administration (Figure 7E). Moreover, miR‐150‐5p expression, which was reduced in CLP mice, was restored after NORAD knockdown (Figure 7F). Western blot analysis further demonstrated that CLP induced increases in cleaved caspase‐3, p‐STAT1/STAT1, and p‐p65/p65, all of which were reversed upon AAV‐sh‐NORAD treatment (Figure 7G). Overall, these results indicate that NORAD is upregulated in lung tissues during sepsis‐induced ALI, and its knockdown mitigates lung injury, inflammation, pulmonary edema, and apoptosis‐related signaling.

## 4 Discussion

Sepsis is a life‐threatening condition associated with dysregulated inflammation, coagulopathy, and multiorgan injury. The lung is particularly vulnerable, and sepsis‐induced ALI—marked by disruption of the pulmonary microvasculature and endothelial barrier—can progress to ARDS, posing a major clinical challenge [25, 26]. Emerging evidence implicates lncRNAs in the pathogenesis of sepsis and its complications. In this study, we identified NORAD as a critical regulator of sepsis‐induced ALI and demonstrated that its inhibitory effects are mediated through the miR‐150‐5p/STAT1 signaling axis.

NORAD has been increasingly recognized as an important regulator in inflammatory diseases. Lei et al. reported elevated NORAD expression in colonic mucosa from patients with ulcerative colitis and in TNF‐α‐stimulated human colonic epithelial cells [27], while another study showed elevated NORAD expression in pulmonary tuberculosis patients, correlating with proinflammatory cytokines IL‐1β, TNF‐α, and IL‐6 [28]. Consistent with these findings, we observed high NORAD expression in LPS‐treated lung epithelial cells, and its silencing suppressed sepsis‐induced inflammatory responses and apoptosis in vitro. LPS, a major endotoxin derived from Gram‐negative bacteria, is widely used to model ALI because of its potent ability to activate inflammatory and oxidative stress pathways in pulmonary cells, thereby recapitulating key pathological features of ALI [29, 30]. Clinically, elevated circulating cytokines including TNF‐α and IL‐6 are strongly associated with disease severity and poor outcomes in sepsis‐induced ALI [3], while excessive activation of pro‐apoptotic mediators such as cleaved caspase‐3 contributes to epithelial cell loss and disruption of the alveolar barrier [5]. In agreement with these observations, our in vivo data showed that NORAD knockdown alleviated lung injury in septic mice, supporting its role as a driver of sepsis‐induced ALI. Notably, a recent study reported that NORAD aggravates septic lung injury through the miR‐155‐5p/TLR6 axis in a rat model, highlighting the involvement of Toll‐like receptor‐mediated innate immune signaling [31]. While both studies identify NORAD as a critical contributor to sepsis‐induced ALI, the downstream mechanisms appear to differ. In contrast to the miR‐155‐5p/TLR6 pathway, which primarily modulates innate immune receptor activation, our findings demonstrate that NORAD promotes lung injury through the miR‐150‐5p/STAT1/NF‐κB axis, a signaling cascade closely linked to transcriptional regulation of inflammation, epithelial barrier dysfunction, and apoptosis. These observations suggest that NORAD may exert pleiotropic and context‐dependent regulatory functions by engaging distinct miRNA‐mediated pathways during sepsis‐induced ALI. While neutrophil infiltration is recognized as a key pathogenic factor in ALI, quantitative assessment of infiltrating neutrophils was not performed in the present study. This limitation has been acknowledged and will be addressed in future investigations.

Next, we identified NORAD as a direct regulator of miR‐150‐5p, suppressing its expression. MiR‐150‐5p acts as a negative modulator of inflammation and apoptosis, as evidenced by its downregulation in LPS‐stimulated lung epithelial cells and its protective effects in other models of sepsis and acute organ injury [32]. Similar protective effects have been reported in septic acute kidney injury, where a miR‐150‐5p agomir reduced LPS‐induced apoptosis in type II alveolar epithelial cells, where miR‐150‐5p overexpression mitigated LPS‐induced A549 cell injury [33]. Consistent with these findings, miR‐150‐5p was downregulated in sepsis and attenuated NORAD‐mediated injury in LPS‐treated lung epithelial cells. Functional rescue experiments confirmed that the inflammatory and apoptotic effects of NORAD knockdown or overexpression are largely mediated through miR‐150‐5p, establishing NORAD as its upstream regulator. Although some NORAD‐associated changes, such as the ~threefold increase in inflammatory cytokines and the relatively small decrease in miR‐150‐5p expression, may appear modest, these molecules act within tightly controlled signaling networks. Even minor perturbations in key lncRNAs or miRNAs can produce amplified downstream responses, influencing multiple targets such as STAT1, NF‐κB, and cleaved caspase‐3, and thereby generating significant functional consequences in lung epithelial injury. This highlights that the observed changes in NORAD and miR‐150‐5p are biologically meaningful in the context of sepsis‐induced ALI. Mechanistically, NORAD promotes lung epithelial apoptosis primarily through suppression of miR‐150‐5p, which in turn activates STAT1 and NF‐κB signaling, leading to increased expression of pro‐apoptotic mediators and inflammatory cytokines. While our study focused on apoptosis, we acknowledge that other forms of regulated cell death, such as necroptosis or ferroptosis, may also contribute and warrant future investigation. Collectively, these results indicate that NORAD exacerbates sepsis‐induced ALI primarily by suppressing miR‐150‐5p and activating downstream STAT1/NF‐κB‐dependent pathways, highlighting its potential as a therapeutic target.

Our further data showed that STAT1 was identified as a direct target of miR‐150‐5p, and its expression was suppressed by miR‐150‐5p overexpression. STAT1, a key component of the JAK/STAT signaling pathway, plays an important role in cytokine signaling and immune regulation [21]. Previous studies have shown that STAT1 activation contributes to ox‐LDL‐induced inflammation via Mettl3 [34], and that STAT1 deletion alleviates LPS‐induced inflammatory responses by reducing IL‐6, IL‐1β, and TNF‐α in ATDC5 cells [35]. Moreover, Bian et al. demonstrated that miR‐150‐5p inhibits atherosclerosis by modulating STAT1‐mediated vascular smooth muscle cell proliferation and migration [36]. Consistent with these findings, our results revealed that NORAD upregulates STAT1 expression by competitively binding to miR‐150‐5p, thereby enhancing sepsis‐induced lung inflammation and apoptosis, which was further confirmed by rescue experiments. Mechanistically, NORAD silencing reduced LPS‐induced phosphorylation of STAT1 and p65 without affecting JAK3 or p‐JAK3, indicating that NORAD primarily modulates inflammatory signaling via the STAT1‐NF‐κB axis rather than through the JAK3 pathway. This observation aligns with evidence that STAT1 activation occurs mainly through JAK1/2 in interferon signaling, whereas JAK3 predominantly mediates γ‐chain cytokine receptor pathways independently of STAT1 [37]. The transcriptional cooperation between STAT1 and NF‐κB provides a mechanistic basis for the reduced NF‐κB activity observed upon STAT1 inhibition, likely due to disrupted promoter co‐occupancy and weakened cytokine feedback [38]. In support of this concept, Fu et al. described a STAT1/HMGB1/NF‐κB cascade driving sustained inflammation and chronic kidney injury, underscoring the role of STAT1 as a central regulator of NF‐κB‐driven inflammatory responses [39].

Taken together, our findings indicate that LPS or CLP exposure induces ALI by promoting inflammation and apoptosis, whereas inhibition of NORAD mitigates these pathological changes. Mechanistically, NORAD acts as a competing endogenous RNA for miR‐150‐5p, leading to upregulation of STAT1 and subsequent activation of the STAT1‐NF‐κB axis, thereby exacerbating sepsis‐induced ALI. These results highlight NORAD as a critical upstream regulator of inflammatory signaling in ALI and suggest its potential as a novel therapeutic target for sepsis‐associated ALI.

## Ethics Statement

All animal procedures were performed in accordance with national guidelines and approved by the Experimental Animal Ethics Committee of Jiujiang No. 1 People's Hospital (Jiangxi, China).

## Conflicts of Interest

The authors declare no conflicts of interest.

## Supporting information


**Figure S1:** All the uncropped protein bands.

## Data Availability

The data that support the findings of this study are available from the corresponding author upon reasonable request.
