# Supplementary figures and images for: LncRNA NORAD Enhances Inflammatory Injury in Sepsis‐Associated Acute Lung Damage Through miR‐150‐5p/STAT1‐Dependent NF‐κB Activation
Source: Kaohsiung J Med Sci. 2026 Mar 3:e70191. Online ahead of print. doi: 10.1002/kjm2.70191 (PMC13399771; doi:10.1002/kjm2.70191)

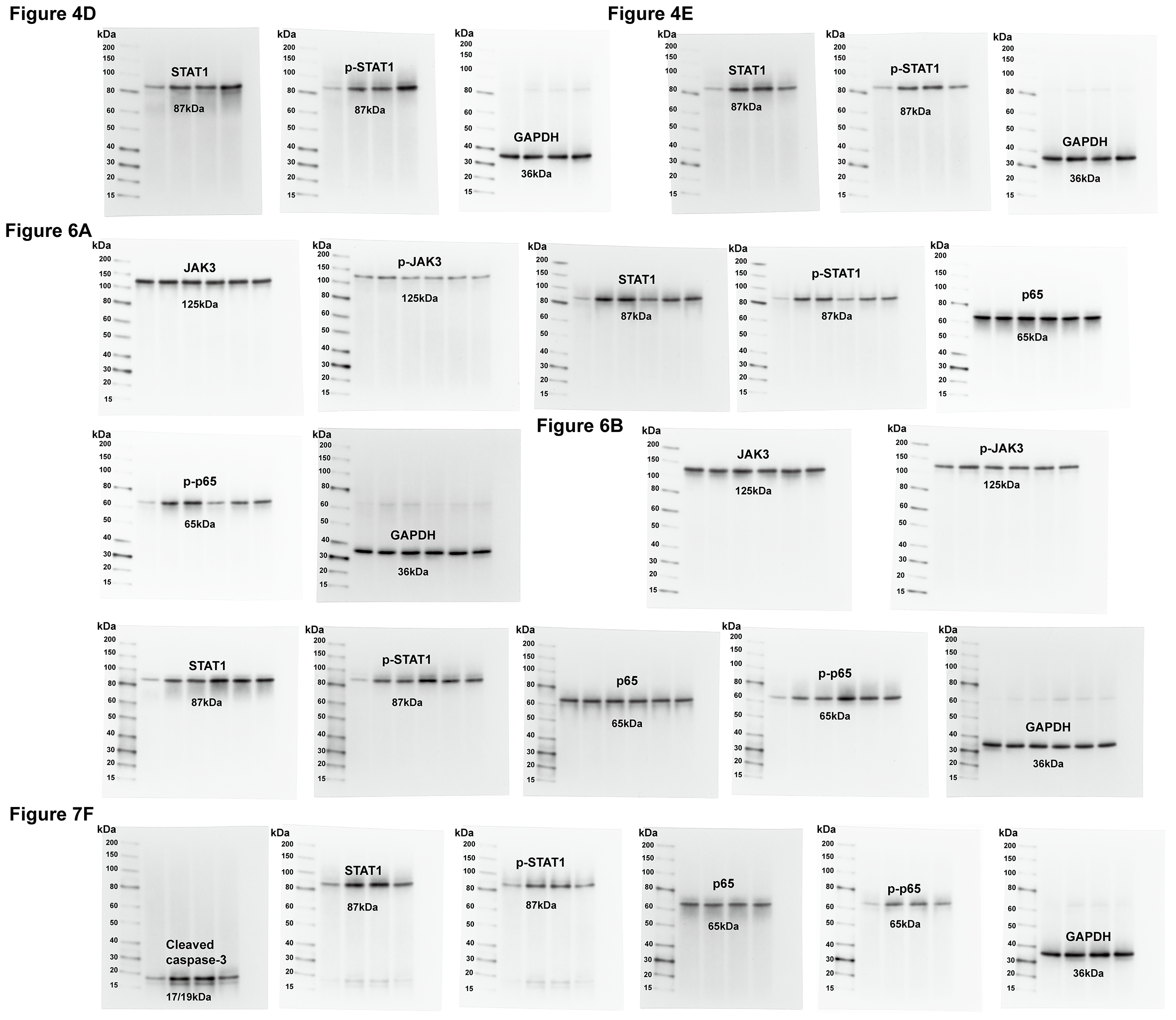

Supplement: Supplementary file 1 — Figure S1: All the uncropped protein bands. [file KJM2-9999-e70191-s001.tif]
